# Is Increased Video Game Participation Associated With Reduced Sense of Loneliness? A Systematic Review and Meta-Analysis

**DOI:** 10.3389/fpubh.2022.898338

**Published:** 2022-05-16

**Authors:** Yan Luo, Michelle Moosbrugger, Daniel M. Smith, Thaddeus J. France, Jieru Ma, Jinxiang Xiao

**Affiliations:** ^1^Department of Physical Education and Health Education, Springfield College, Springfield, MA, United States; ^2^Department of Sport Management and Recreation, Springfield College, Springfield, MA, United States; ^3^Sports Business School, Beijing Sport University, Beijing, China; ^4^Sports Coaching College, Beijing Sport University, Beijing, China

**Keywords:** video gaming, online gaming, psychological wellbeing, mental health, electronic game

## Abstract

The purpose of this systematic review was to evaluate the existing evidence in literature addressing the relationship between video game participation and loneliness. The following databases were searched on October 2, 2021: Medline, Psychinfo, SportDiscus, Web of Science, and CINAHL. The risk of bias of cross-sectional study was assessed by using the Joanna Briggs Institute (JBI) critical appraisal checklist for analytical cross-sectional studies with attrition bias added for longitudinal studies. The results of all included studies were synthesized using narrative synthesis. Meta-analysis was utilized to synthesis the findings of the studies that had sufficient degree of statistical and methodological homogeneity. Eighteen studies were included in this systematic review, which comprised of 20,372 participants. The narrative synthesis showed mixed findings on the relationship between video game participation and loneliness. Meta-analysis that was conducted to nine cross-sectional studies revealed that video game participation was positively and weakly associated with loneliness (*r* = 0.10, 95% CI = 0.03–0.17). Both cross-sectional and longitudinal studies demonstrated serious risk of bias with the addition of serious inconsistency of findings from cross-sectional studies. The existing literature is equivocal in terms of making a definitive judgment on the association between video game participation and loneliness. PROSPERO registration number: CRD42021283025.

**Systematic Review Registration:** https://www.crd.york.ac.uk/PROSPERO, identifier: CRD42021283025.

## 1. Introduction

Due to the increased accessibility of personal computers, video game (VG) playing has risen to prominence as one of the most popular recreational activities worldwide in recent years. According to Newzoo, there are approximately three billion video gamers worldwide in 2021 (1). This figure has grown at a constant rate of 5.6% on a yearly average basis (1). One of the major reasons people choose to engage in VG is its entertaining and enjoyable nature. In addition, it has been found that playing VG is positively associated with favorable psychological outcomes (2–4). However, VG participation can also be highly addictive (5), resulting in physical (6), and mental fatigue (7), depressive symptoms (8), anxiety (5), and loneliness (9). As an increasing number of individuals embark on playing VG, it is critical to understand its consequences on certain areas of mental health, therefore giving evidence for policymakers and facilitating the implementation of effective interventions to address this issue.

Loneliness is defined as an internal unpleasant state which arises from the perception of a lack of desired interpersonal relationships (10). Loneliness is one of the factors that are thought to be harmful to an individual's overall well-being. Previous researchers have established a link between loneliness and a variety of adverse outcomes. Individuals who have an increased sense of loneliness are more likely to experience daytime sleepiness (11), have a lower degree of life satisfaction (12), exhibit more depressive symptoms (13), and even fantasize about suicide (14). It is critical to understand the underlying factors that contribute to a heightened sense of loneliness in order to avoid its detrimental effects.

The Basic Psychological Needs (BPN) theory postulates that humans are intrinsically prone to psychological growth and integration (15, 16). This intrinsic declination of psychological growth automatizes us to seek for self-perceived positive experiences through actively interacting with environments. The psychological growth process is characterized by three factors: autonomy, competence, and relatedness (16). Relatedness refers to a sense of connection and belonging (16). The natural proclivity toward relatedness can be considered as a natural defense against loneliness. It is plausible to assume that people are inherently predisposed to seek relatedness and avoid loneliness.

Stress is a negative affective state related with the believed inability to overcome adversity (17). Coping is a term that refers to cognitive and behavioral attempts made to meet certain external or internal demands that are seen to be taxing or surpassing the individual's resources (17). According to Compas et al. (18), there are two distinct sorts of coping strategies: engagement coping and disengagement coping. While engagement coping strategy means proactively confronting and overcoming a stressor, disengagement coping strategy involves withdrawing from or avoiding exposure to a stressor (18). According to stress-coping theory, individuals may choose to withdraw from a stressor by engaging in maladaptive behaviors when their previous coping approach fails or the coping behavior is potentially addictive (4). Heavy VG players are likely to use VGs as a way to escape from negative feelings (19). Given that numerous studies have demonstrated that VG engagement could be seriously addictive (20), it is possible that loneliness acts as a stressor, resulting in increased level of VG participation.

Social presence (SP) was originally defined as “the degree of salience of the other person in the interaction and the consequent salience of the interpersonal relationships” [(21), p. 65]. Today, in the context of virtual environments, the definition of SP in a virtual context was “a psychological state in which virtual social actors are experienced as actual social actors in a sensory or non-sensory way” [(22), p. 45]. According to these definitions, SP should be considered a critical factor in reducing loneliness in physical or virtual interactions. A stronger sensation of SP indicates that one actor observes the other and their interpersonal relationship more prominently, thus establishing a stronger sense of relatedness. The level of SP in any form of communication is determined by three essential components: co-presence, psychological involvement, and behavioral engagement.

Co-presence refers to “the degree to which the observer believes he/she is not alone and secluded, their level of peripheral or focal awareness of the other, and their sense of the degree to which the other is peripherally or focally aware of them” [(23), p. 247]. Psychological involvement refers to “the degree to which the observer allocates focal attention to the other, empathically senses or responds to the emotional states of the other, and believes that he/she has insight into the intentions, motivation, and thoughts of the other” [(24), p. 2]. Behavioral engagement refers to “the degree to which the observer believes his/her actions are interdependent, connected to, or responsive to the other and the perceived responsiveness of the other to the observer's actions” [(24), p. 2]. When playing VGs, players are aware of each other, and they need to understand each other's intentions and thoughts in order to take joint efforts to achieve the same goal. Therefore, VG provides players the opportunity to experience the feeling of SP *via* the copresence, psychological involvement, and behavioral engagement mechanism. This sense of presence will likely to be converted into a resource that satisfies the sense of relatedness and alleviates loneliness.

Though many prior researchers have studied the association between loneliness and VG participation, there has been no systematic review in this field. A systematic review enables researchers to evaluate and synthesize all available evidence on a certain topic (25). One notable feature of this review approach is that it establishes a set of thorough and rigorous procedural standards for researchers to follow so that the results are more likely to be replicated by others (26). As previously stated, loneliness has a plethora of detrimental mental repercussions. By combining prior evidence, researchers and practitioners can be informed with more robust information on the topic, influencing future studies and intervention initiatives. The purpose of this systematic review was to evaluate the existing evidence regarding the relationship between VG participation and loneliness.

## 2. Method

### 2.1. Protocol and Eligibility Criteria

The present study was pre-registered on the International Prospective Register of Systematic Reviews (PROSPERO, registration number CRD42021283025). The present study was conducted and reported according to the Preferred Reporting Items for Systematic Reviews and Meta-Analyses guidelines [PRISMA, (27)]. The Population, Interventions (exposure), Comparisons, Outcomes, and Study design framework [PICOS, (28)] was used to identify important research variables, and to inform search strategy.

No restrictions were put on the population, comparison, and study design. Exergames, including virtual reality (VR) games, were omitted from this study since playing them may result in an increased amount of SP as compared to traditional VGs (29). The outcome was restricted to subjective psychological loneliness rather than objective social isolation since studies have found the feeling of loneliness can occur with the absence of social isolation (30). Other eligibility criteria included: (1) measurement of VG participation and loneliness; (2) examination of the relationship between VG participation and loneliness; (3) peer-reviewed articles; (4) employed quantitative methodology; (5) written in English; (6) not scale development or validation study; (7) not gray literature, abstracts, theses, and conference proceedings.

### 2.2. Information Sources and Search Strategy

A search of titles, abstracts, and key words was conducted on October 2, 2021 for the following five databases: Medline, Psychinfo, SportDiscus, Web of Science, and CINAHL. No restrictions were placed on publication date. Additional studies that met the inclusion criteria were identified by searching reference lists.

Search parameters (key words) were developed based on the practice of previous studies (31, 32). In addition, the primary researcher posted the search parameters online and asked for careful examination of them from peers. The following key words were used to search in the database: (“electronic gam*” OR “video gam*” OR “online gam*” OR esport* OR “digital gam*” OR “mobile gam*” OR “phone gam*” OR “electronic sport*” OR “computer gam*” OR “internet gam*”) AND lonel*.

### 2.3. Study Selection

Results of the search were imported into Zotero (version 5.0) where duplicates were removed. The screening procedure was done by the primary investigator and confirmed by one of the co-authors. Consensus was established *via* discussion. Titles and abstracts were screened against the eligibility criteria, followed by retrieving the full texts of potentially eligible articles. The retrieved full articles were scrutinized to determine their suitability for inclusion. When full texts were not available, the articles were requested from the author *via* email.

### 2.4. Data Extraction

Data extraction form was created in Microsoft Excel. The extraction process was completed by the primary investigator and checked for accuracy by one of the co-authors. The following information was extracted from each article: author, publication year, study design, country, participant characteristics, sample size, loneliness measure, VG participation measure, game name, effect size indicators (when applicable).

### 2.5. Risk of Bias and Quality of Evidence

The risk of bias of cross-sectional studies was evaluated by using the Joanna Briggs Institute (JBI) critical appraisal checklist for analytical cross-sectional studies (33). This tool used eight criteria to evaluate the overall methodological quality of a study. The criteria include: sample inclusion criteria; description of subjects and settings; valid and reliable measure of exposure; objective and standard measure of condition; identifying confounding factors; strategies to deal with confounding factors; valid and reliable measure of outcome; and appropriate statistical analysis (33). For observational longitudinal studies, the attrition bias (incomplete follow-up) from the Cochrane Handbook (34) was added to the previous risk of bias list. The following options were used to answer each criterion: yes (satisfied), no (not satisfied), unclear, and na (not applicable). The overall risk of bias of each study was rated on high, fair, and low (coded 2, 1, 0 for reliability analysis, respectively). The risk of bias was assessed independently by the primary investigator and one of the co-authors. Krippendorff's α was used to quantify interrater reliability of the overall risk of bias score which runs from 0 (prefect disagreement) to 1 (perfect agreement). The inconsistencies of each criterion and overall risk of bias scores between two authors were addressed by discussion.

The Grading of Recommendations Assessment, Development, and Evaluation (GRADE) framework (35) was used to evaluate the overall quality of evidence regarding the association between loneliness and VG participation. The GRADE approach enables researchers to describe the level of confidence in an estimated effect on a quality continuum (high, moderate, low, or very low). While high quality indicates that future studies are unlikely to alter the estimated effect, very low quality indicates that the estimated effect is very uncertain. Only randomized controlled trials begin as high-quality evidence. All other study designs are first graded as low-quality evidence. If there is a significant risk of bias, inconsistency of results, indirectness of evidence, imprecision, or reporting bias, the quality of evidence is downgraded. The quality of evidence may be raised if there is a very high effect size, a dose-response gradient, or if all potential biases would result in a decrease in the estimated effect. The primary investigator evaluated the quality of evidence across studies.

### 2.6. Synthesis of Results

The results of all included studies were synthesized using narrative synthesis. Additionally, meta-analysis was used to synthesize the results of the studies that were deemed having sufficient degree of statistical and methodological homogeneity. Meta-analysis with random-effect model was performed using R (36). The reason for using a random-effect model was because the true effect size was expected to vary due to the diversity of participants' background. The aggregated effect size is considered significant if the 95% confidence interval (95% CI) does not contain 0. *I*^2^ was used to assess the heterogeneity of effect size across studies. The *I*^2^ value higher than 50% indicates a substantial heterogeneity across studies (37). When a study's 95% CI was outside of the 95% CI of the overall effect size on either side, it was considered an outlier (37). Ninety-five percent of prediction interval (95%PI) was used to estimate the range of effect size that 95% future studies will fall. Publication bias was assessed by the following ways. A funnel plot was generated by graphing each study's effect sizes against standard errors. Symmetric distribution of effect sizes around the overall effect size shows that there is no substantial publication bias (38). Egger's regression test was used to assess publication bias, with a regression intercept significantly different than zero indicating considerable publication bias (39). Missing study effect sizes were imputed using the trim-and-fill approach (40). If the adjusted overall effect size is significantly different from the original overall effect size, publication bias exists.

## 3. Results

### 3.1. Study Selection

**Figure 1:**
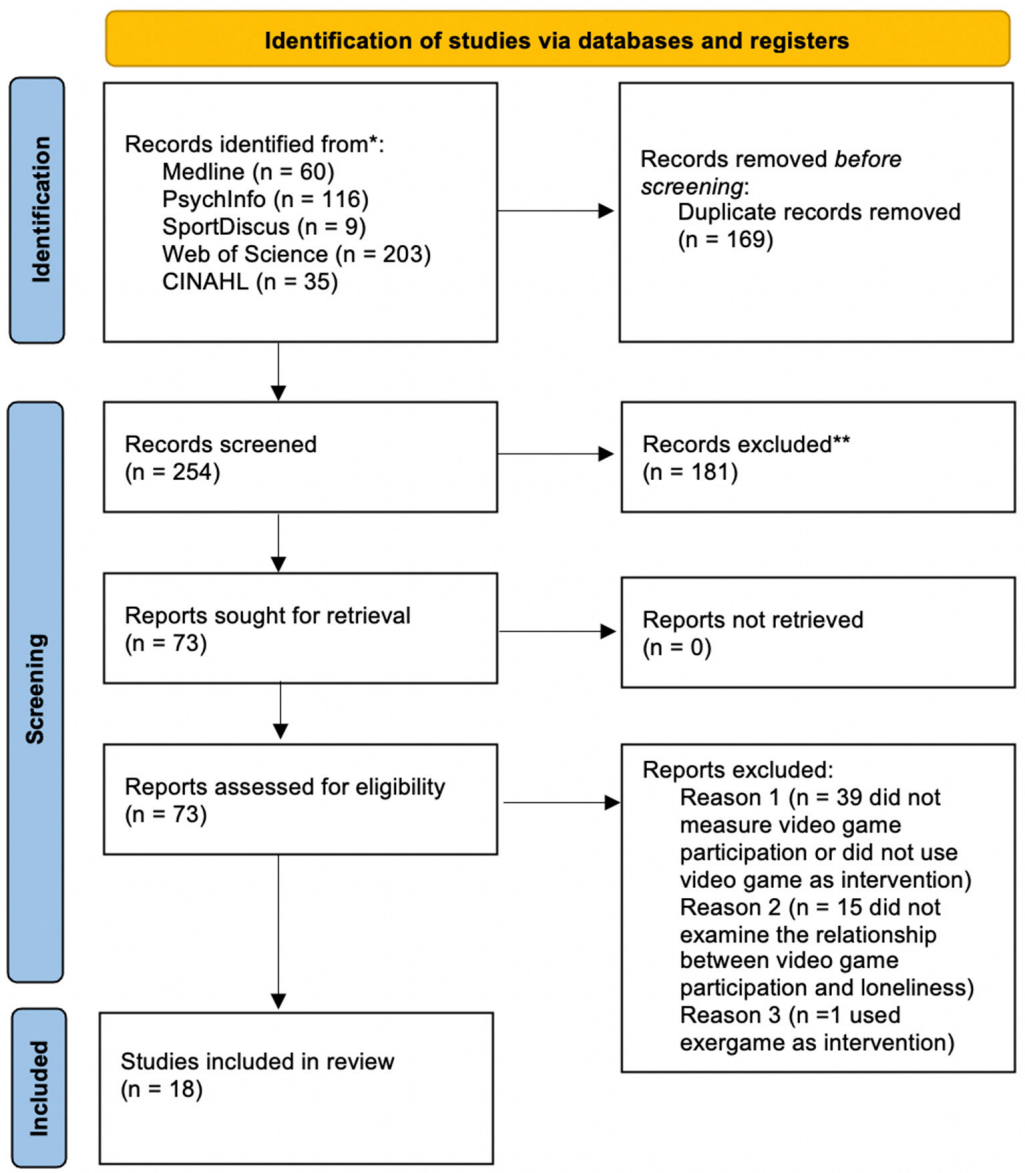
Flow chart of the study selection process.

Four hundred and twenty-three articles were identified through database searches. After deleting 169 duplicates, the titles and abstracts were reviewed for the remaining 254 articles. One hundred and eighty-one articles were omitted from consideration due to their deemed inapplicability to this study. The full text of 73 article were retrieved. Thirty-nine articles were omitted owing to a lack of VG participation measurement or did not use VG as intervention. Fifteen articles were excluded due to failing to examine the association between VG participation and loneliness. One article was excluded because of the use of exergame (41). The current review consists of 18 articles. Figure 1 shows the flow chart of the study selection process.

### 3.2. Study Characteristics

The 18 articles included 20,372 participants from a variety of countries, including the United States (*n* = 3), China (*n* = 3), Netherlands (*n* = 3), South Korea (*n* = 2), Sweden (*n* = 1), Canada (*n* = 1), Finland (*n* = 1), Norway (*n* = 1), Germany (*n* = 1), Spain (*n* = 1), and a global study (*n* = 1). The sample size ranged from 151 to 5,000. One study did not specify the exact number of participants (42). While males comprised of 74.24% of the total participants (*n* = 15,124), females accounted for 25.59% of the total participants (*n* = 5,214), and 0.17% (*n* = 34) of the participants did not declare gender information. While 16 studies employed a cross-sectional methodology, two studies used longitudinal panel design. The characteristics of each study were summarized in Table 1.

**Table 1:** Study characteristics.

**References**	**Study design**	**Country**	**Sample characteristics**	**Sample size**	**Loneliness measure**	**Video game participation measure**	**Game genre**
Zhou and Leung (43)	Cross-sectional	Mainland, China	Undergraduate college students aged from 18 to 22; male = 32.2%; female = 67.8%	342	Revised UCLA 20-item Loneliness Scale. Cronbach's α = 74	A composite measure of three questionnaire questions. Cronbach's α = 0.74	Happy Farm
Yang and Liu (44)	Cross-sectional	United States	Participants aged from 18 to 58 with a mean age of 30.71 (SD = 7.77); male = 55%; female = 45%	262	UCLA 20-item Loneliness Scale. Cronbach's α = 0.98	One questionnaire question	Poke'mon Go
Wu et al. (45)	Cross-sectional	Macao, China	Participants aged between 18 to 30 with a mean age of 22.7 (SD = 2.73); female = 55%; male = 45%	165	Short form of the UCLA Loneliness Scale. Cronbach's α = 0.80. Loneliness was recoded into connectedness	One questionnaire question	NA
Visser et al. (46)	Cross-sectional	Netherlands	High school students and WoW players aged from 11 to 20 years with a mean age of 14.8 (SD = 1.8). Two-hundred and forty one were WoW players (30.5%) and 548 were not (69.5%); male = 50.70%; female = 49.30%.	789	Eight items from the UCLA Loneliness Scale. Cronbach's α = 0.76	Did not specify	WoW
Verheijen et al. (47)	Cross-sectional	Dutch	High school students with a mean age of 14.07 (SD = 1.29); male = 66.5%; female = 33.5%	705	Loneliness and Aloneness Scale for Children and Adolescents. Cronbach's α = 0.90	Two questionnaire questions	NA
Sundberg (48)	Cross-sectional	Sweden	The experimental group included people with Autism and ASD. The control group included university students and different Facebook groups. Participants aged between 14 and 69 years with a mean age of 26.68 (SD = 10.78); female = 56.95%; male = 43.05.	151	Short version of the original UCLA loneliness scale. Cronbach's α for ASD gamers = 0.84. Cronbach's α for ASD non-gamers = 0.85. Cronbach's α for control gamers = 0.85. Cronbach's α for control non-gamers = 0.78	One questionnaire question	NA
Snodgrass et al. (49)	Cross-sectional	Globe	Over two thirds of the participants were students with a mean age of 21 (SD = 6); male = 90.40%; female = 9.60%	3,629	3-item loneliness scale. Cronbach's α = 0.82. Loneliness was measured as an indicator of social distress	One questionnaire question	NA
Shen and Williams (42)	Cross-sectional	United States	EQII players with a mean age of 31.16; male = 80.80%; female = 19.20%	Around 5,000	20-item UCLA Loneliness Scale. Cronbach's α = 0.92	EQII time was measured by finding the logs generated by the game servers; Time spent on games other than EQII was measured by one questionnaire question”	EQII
Punamäki et al. (50)	Cross-sectional	Finland	222 were fourth graders with a mean age of 10.27 (SD = 0.47) and 256 were seventh graders with a mean age of 13.28 (SD = 0.46); female = 54.40%; male = 45.6%	478	Six-item Children's Loneliness Scale. Cronbach's α = 0.77	A composite measure of three questionnaire questions. Cronbach's α = 0.78	na
Myrseth et al. (51)	Cross-sectional	Norway	Participants in a gambling and gaming study with a mean age of 19.5 years (SD = 1.0); male = 80.33%; female = 19.67%	1,017	Robert's 8-item UCLA Loneliness Scale. Cronbach's α = 0.70	One questionnaire question	na
Martončik and Lokša (52)	Cross-sectional	United States	WoW internet message board users. Over two thirds of the participants came from the United States. male = 88.20%; female = 11.80%	161	UCLA 20 item Loneliness Scale. ω = 0.95 for real-world loneliness. ω = 0.95 for online loneliness.	One questionnaire question	WoW
Koban et al. (9)	Cross-sectional	NA	Video game forums users aged from 18 to 62 with a mean age of 25.22 (SD = 6.68); male = 85.88%; female = 13.19%	3,655	Short version of the UCLA Loneliness Scale. Cronbach's α = 0.87	A composite measure of two questionnaire questions	NA
Jeong et al. (53)	Cross-sectional	South Korea	Elementary 6th graders; male = 51%; female = 49%	944	Six-item UCLA loneliness scale. Cronbach's α = 0.91	One questionnaire question	NA
Cheung et al. (54)	Cross-sectional	Hong Kong	Secondary school students; females = 48.3%; males = 51.7%; 0.6% (4) of the participants aged below 12; 57% (378) aged from 12 to 14; 40.3% (267) aged from 15 to 17 years; 2.1% (14) aged 18 and above	632	Third version of the revised Chinese UCLA loneliness Scale. Cronbach's α = 0.90	One questionnaire question	NA
Buiza-Aguado et al. (55)	Cross-sectional	Spain	Students aged from 12 to 18 years with a mean age of 15.6 (SD = 2.7); male = 55.80%; female = 44.20%	708	Five questions from the 20-item UCLA Loneliness Scale	Two questionnaire questions	NA
Ahn and Shin (56)	Cross-sectional	Korea	Participants aged from 19 to 39 with a mean age of 29.02 (SD = 5.22) in an online survey research pool; male = 50.00%; female = 50.00%	300	Modified connectedness subscale of the Korean version of revised UCLA Loneliness Scale. Cronbach's α = 0.89	One questionnaire question	NA
Lemmens et al. (57)	Longitudinal	Dutch	Secondary school students aged from 11 to 17 with a mean age of 13.9 (SD = 1.4); male = 69.98%; female = 30.02%	543	Five items from the UCLA 20-item loneliness scale. Cronbach's α = 0.90 in the first wave and α = 0.91 in the second wave.	Two questionnaire questions	NA
Kowert et al. (58)	Longitudinal	Germany	Adolescents aged from 14 to 18 (*N* = 110); young adults aged from 19 to 39 (*N* = 358); and older adults aged from 40 and over (*N* = 423); male = 57.01%; female = 42.99%.	891	Two items from the UCLA loneliness scale. Cronbach's α = 0.66 in the first wave and α = 0.59 in the second wave	One questionnaire question	NA

*WoW, world of Warcraft; na, not applicable; ASD, autism spectrum disorder; EQII, everquest II*.

### 3.3. Risk of Bias

The risk of bias of included studies ranged from fair (*n* = 15) to high (*n* = 3). Krippendorff's α was 0.83. The primary grounds for rating the studies as having a fair risk of bias were: (1) the majority of studies, with the exception of two (9, 45), did not explicitly describe their sample inclusion criteria; (2) all studies asked participants to recall their level of VG participation within a certain time period in the past (one study used the logs generated by the game server to measure Everquest II usage, but asked participants to recall their VG participation time for other VGs (42). The validity and reliability of this type of assessment was unclear. Potential recall bias of this assessment approach was well-documented (59). In addition to these common drawbacks, three studies were considered as having a high risk of bias for the following reasons: (1) one study reported an impossible reliability alpha of 74 for loneliness measure (43); (2) one study did not report the reliability of loneliness scale (55); (3) one study had a significant likelihood of attrition bias (294 of the initial 4,500 participants completed all study waves) and the Cronbach's alpha was reported as 0.59 for the loneliness scale at the second wave of the study (58). Cronbach's alpha <0.70 should be considered unacceptable according to Nunnally (60). Table 2 contains thorough information on the risk of bias of included studies.

**Table 2:** Risk of bias of included studies.

**References**	**Inclusion criteria**	**Detailed description of subjects and setting**	**Valid and reliable measure of exposure**	**Objective and standard measure of condition**	**Confounding factors identified**	**Strategies to deal with confounding factors**	**Valid and reliable measure of outcome**	**Appropriate statistical analysis**	**Low attrition bias**	**Overall**
Zhou and Leung (43)	N	Y	U	NA	Y	Y	N	Y	NA	High
Yang and Liu (44)	N	N	U	NA	Y	Y	Y	Y	NA	Fair
Wu et al. (45)	Y	Y	U	NA	N	NA	Y	y	NA	Fair
Visser et al. (46)	N	Y	U	NA	N	NA	Y	Y	NA	Fair
Verheijen et al. (47)	N	Y	U	NA	Y	Y	Y	Y	NA	Fair
Sundberg (48)	N	Y	U	Y	Y	Y	Y	Y	NA	Fair
Snodgrass et al. (49)	N	Y	U	NA	N	NA	Y	Y	NA	Fair
Shen and Williams (42)	N	Y	y & U	NA	Y	Y	Y	Y	NA	Fair
Punamäki et al. (50)	N	Y	U	NA	N	NA	Y	Y	NA	Fair
Myrseth et al. (51)	N	Y	U	NA	N	NA	Y	Y	NA	Fair
Martončik and Lokša (52)	N	Y	U	NA	Y	Y	Y	Y	NA	Fair
Koban et al. (9)	Y	Y	U	na	N	NA	Y	Y	NA	Fair
Jeong et al. (53)	n	y	U	NA	N	NA	Y	Y	NA	Fair
Cheung et al. (54)	N	Y	U	NA	N	NA	Y	Y	NA	Fair
Buiza-Aguado et al. (55)	N	Y	U	NA	N	NA	U	Y	NA	High
Ahn and Shin (56)	N	Y	U	NA	Y	Y	Y	Y	NA	Fair
Lemmens et al. (57)	N	Y	U	NA	N	Na	Y	Y	Y	Fair
Kowert et al. (58)	N	Y	U	na	y	Y	N	Y	N	High

*N, no (not satisfied); Y, yes (satisfied); U, unclear; NA, not applicable; Y & U, partially satisfied*.

### 3.4. Results of Synthesis

Of the 16 cross-sectional studies, six studies found a significant and positive relationship between VG participation and loneliness (9, 43, 45, 49, 51, 53). Zhou and Leung (43) found this relationship specifically for Happy Farm players and found loneliness significantly predicted Happy Farm use when controlling for gender, age, school year, family income, gaming motivations, leisure boredom, self-esteem, play place, and play history. Wu et al. (45) recoded loneliness into connectedness and found a significant and negative relationship between VG participation and connectedness.

Seven studies did not find a significant relationship between VG participation and loneliness (44, 46, 47, 50, 52, 54, 55). When controlling for gender and age, neither VG participation (47) or Pokemon Go use (44) predicted loneliness. Two studies examined the relationship between World of Warcraft (WoW) use and loneliness (46, 52). Visser et al. (46) found no significant difference in loneliness between WoW players and non-WoW players, whereas (52) found no significant difference in online and real-world loneliness between people who played different amount of WoW. No significant interaction was found between the type of the world (real or online) and WoW use for loneliness (52). Buiza-Aguado et al. (55) found no significant difference in loneliness between heavy gamers and casual gamers.

Three studies found mixed results on the relationship between VG participation and loneliness (42, 48, 56). Sundberg (48) found no difference in loneliness between players who played different amount of game in control groups, but significant difference in loneliness between players was found in autism spectrum disorder (ASD) groups. ASD players who played for less than an hour a day experienced less loneliness than those who played 2–3 h a day and 3–5 h a day, but not those who never play, those who played 1–2 h a day or those who played more than 5 h a day (48). Shen and Williams (42) found that loneliness was not significantly associated with Everquest II use, but was significantly associated with other VG participation. Both Everquest II use and other VG participation predicted each other while controlling for gender, age, depression, and extroversion (42). Ahn and Shin (56) found that VG participation was not significantly associated with connectedness, but predicted connectedness after adding face-to-face communication as a covariate. An indirect effect of VG participation on connectedness through perspective taking was also found (56).

Among the two longitudinal studies, Kowert et al. (58) found that VG participation was significantly associated with loneliness for people aged 19–39 years but not for those aged 14–18 years and above 40 years. VG participation and loneliness did not significantly predict each other 1 year later across all age groups (58). Lemmens et al. (57) discovered that present VG participation was not associated with present loneliness and loneliness 6 months later. VG participation 6 month later was not associated with present loneliness and loneliness 6 months later (57).

Meta-analysis was conducted to nine cross-sectional studies that used similar measurement of VG participation and loneliness and consistently used Pearson's r as the effect size indicator. The reasons for the exclusion from the meta-analysis of the other seven cross-sectional studies were: (1) one study recoded loneliness into relatedness (45); (2) one study measured connectedness instead of loneliness (56); (3) four group comparison studies (46, 48, 52, 55); (4) one study did not report exact sample size (42).

**Figure 2:**
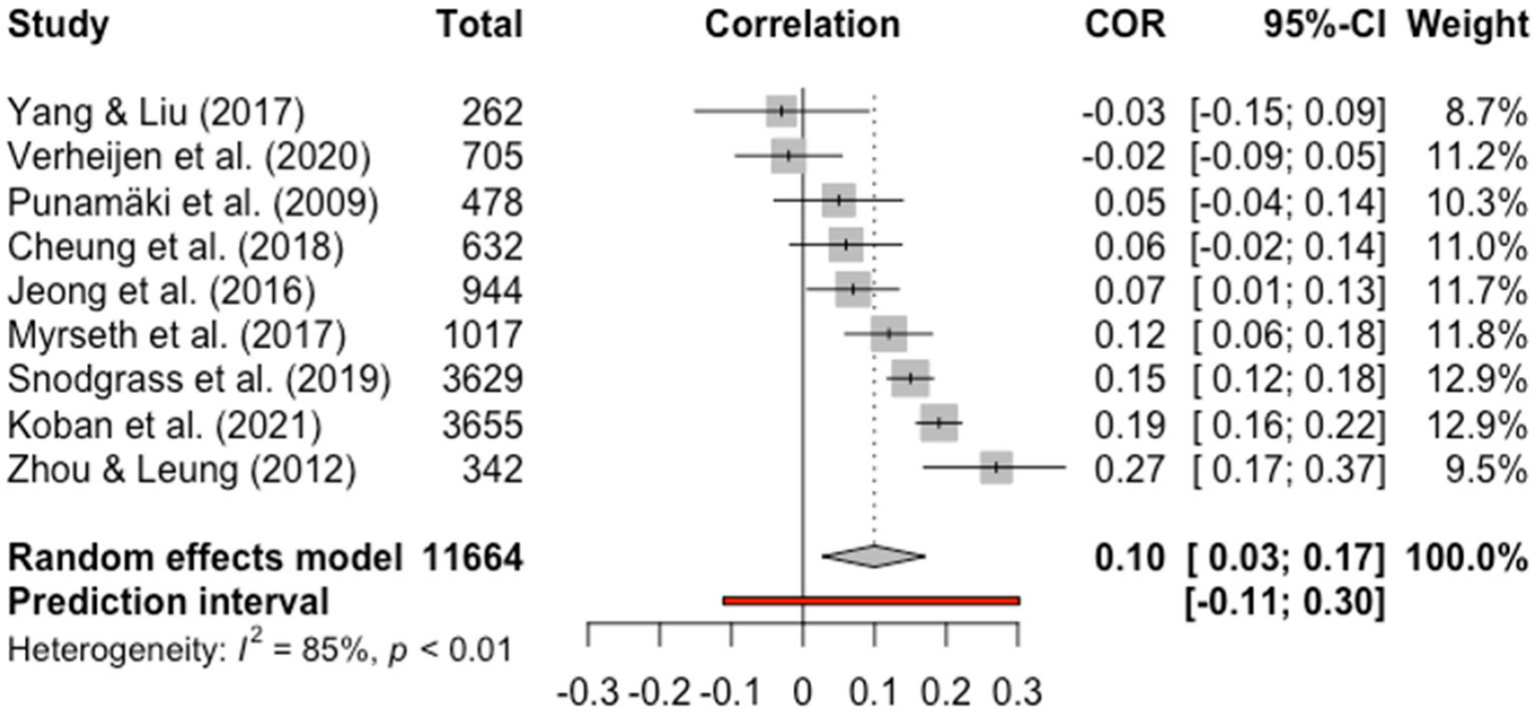
Forest plot.

Through the meta-analysis, VG participation was found to be positively and weakly associated with loneliness [*r* = 0.10, 95% CI = 0.03–0.17, 95%PI = −0.11 to 0.30]. The forest plot in Figure 2 shows the effect sizes (Pearson's *r*) and 95% CI for each study, as well as the overall effect size estimated using the random effects model. The estimated between-study heterogeneity was τ^2^ = 0.007 (95% CI = 0.003–0.034), with an *I*^2^ value of 85.4% (95% CI = 74.2–91.8%). Despite the high degree of heterogeneity in effect sizes among studies, no study was detected as an outlier, as the 95% CI for all studies overlapped with the 95% CI of the overall estimated effect size.

### 3.5. Sensitivity Analysis

After removing an article which has a high degree of risk of bias from the meta-analysis, the results remained stable (*r* = 0.08, 95% CI = 0.02–0.15, τ^2^ = 0.005, 95% CI = 0.002–0.024, *I*^2^ = 85.4%, 95% CI = 73.1–92.0%).

### 3.6. Publication Bias

**Figure 3:**
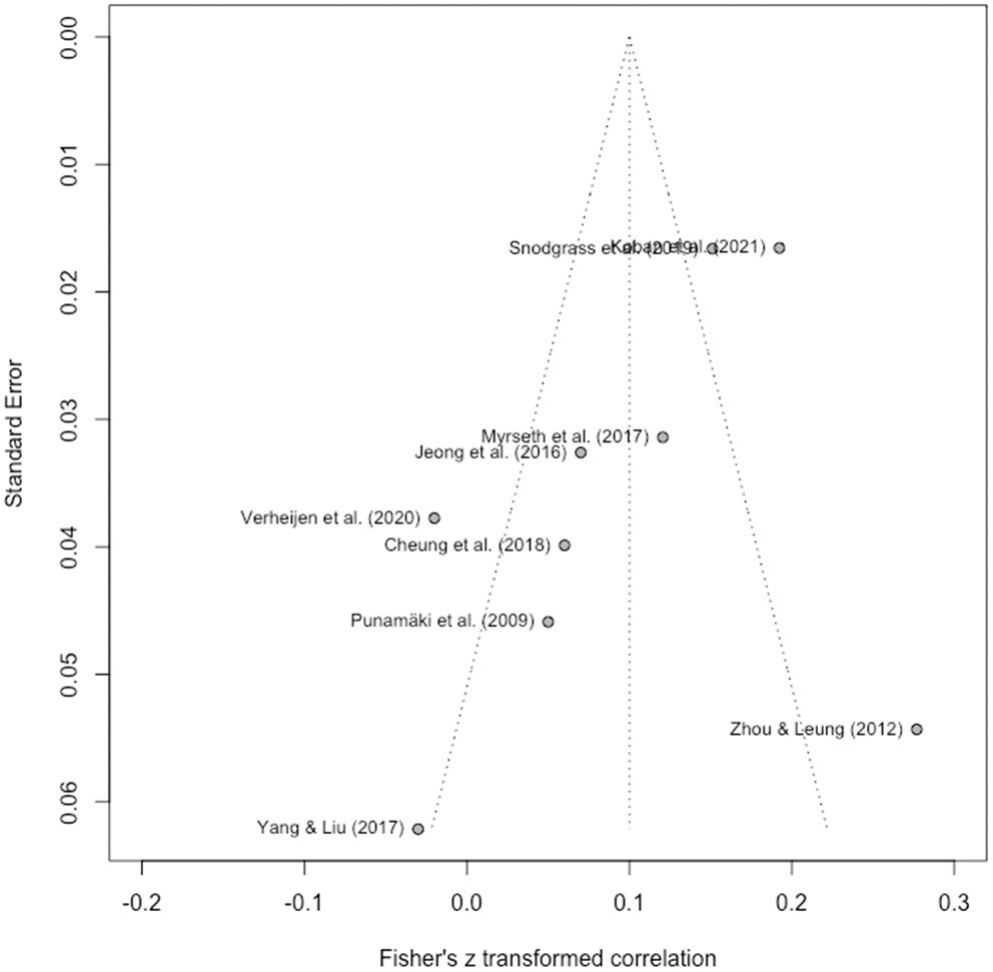
Funnel plot.

Publication bias test was done for all the studies included in the meta-analysis. The funnel plot in Figure 3 demonstrates a slight degree of asymmetry, with all studies with low standard errors demonstrating a greater effect than the overall effect, and only one study with a moderate standard error demonstrating a greater effect than the overall effect. Egger's regression test revealed a non-significant intercept [β = −3.44, *t*_(7)_ = −2.04, *p* = 0.08]. The trim-and-fill approach resulted in a point estimate of *r* = 0.148 (95% CI = 0.068–0.225). Taken together, these analyses revealed no indication of considerable publication bias.

### 3.7. Quality of Evidence

The quality of evidence was presented in Table 3. Overall, the quality of evidence was graded very low for both cross-sectional and longitudinal studies. The reasons for downgrading the quality of evidence for cross-sectional studies were: (1) fair or high risk of bias; (2) inconsistent findings across studies. The quality of evidence for longitudinal studies was downgraded because of fair or high risk of bias.

**Table 3:** Quality of evidence.

		**Quality assessment**						
**Number of participants (number of studies)**	**Design**	**Risk of bias**	**Inconsistency**	**Indirectness**	**Imprecision**	**Reporting bias**	**Absolute effect**	**Quality**
18,938 (16)	Cross-sectional	Serious risk of bias	Serious inconsistency	No serious indirectness	No serious imprecision	None	Significant findings:	Very low
							Loneliness was significantly associated with game use [*r* = 0.15, *p* < 0.01, (49); *r* = 0.12, *p* < 0.05, (51); *r* = 0.19, *p* < 0.01, (9); *r* = 0.07, *p* < 0.05, (53)] and Happy Farm use [*r* = 0.27, *p* < 0.001, (43)];	
							Connectedness was significantly associated with game use [*r* = −0.38, *p* < 0.01, (45)];	
							Mixed findings:	
							No significant difference in loneliness was found in control groups that played different amount of game [*F*_(5, 60)_ = 0.340, *p* = 0.887], whereas significant difference in loneliness was found in ASD groups [*F*_(4, 79)_ = 2.564, *p* = 0.033, (48)];	
							Loneliness was not significantly associated with EQII use [*r* = 0.02, *p* > 0.05], but was significantly associated with other game use [*r* = 0.10, *p* < 0.05, (42)];	
							Connectedness was not significantly associated with game use [*r* = −0.06, *p* > 0.05]; Game use significantly predicted connectedness [β = −0.13, *p* < 0.05] after adding face-to-face communication as a covariate. An indirect effect of game use on connectedness through perspective taking was found [*p* < 0.05, (56)].	
							Null findings:	
							Loneliness was not significantly associated with game use [*r* = 0.05, *p* >0.05, (50); *r* = 0.06, *p* >0.05, (54); *r* = −0.02, *p* >0.05, (47)] and Poke' mon Go use [*r* = −0.03, *p* >0.05, (44)].	
							No significant difference in loneliness was found between WoW players and non-WoW players [*t*_(779)_ = 1.62, *p* >0.05, (46)];	
							No significant difference in online world loneliness [*F*_(3, 112)_ = 0.903, *p* = 0.442] and real world loneliness was found [*F*_(3, 112)_ = 2.270, *p* = 0.084] for groups that played different amount of WoW (52);	
							No significant difference in loneliness was found between heavy gamers and casual gamers [*p* >0.05, (55)].	
1,434 (2)	Longitudinal	Serious risk of bias	No serious inconsistency	No serious indirectness	No serious imprecision	None	Game use was significantly associated with loneliness for people aged 19–39 years (*r* = 0.21, *p* < 0.01) but not for those aged 14–18 years and above 40 years (*p* >0.05). Game use did not significantly predict loneliness 1 year later and vice versa across all age groups [*p* >0.05, (58)];	Very low
							Present game use was not associated with present loneliness (*r* = −0.07, *p* >0.05) and loneliness 6 months later (*r* = −0.07, *p* >0.05); Game use 6 month later was not associated with present loneliness (*r* = −0.01, *p* >0.05) and loneliness 6 months later [*r* = −.01, *p* >0.05, (57)].	

*WoW, world of Warcraft; ASD, autism spectrum disorder; EQII, everquest II*.

## 4. Discussion

This systematic review aimed to evaluate the relationship between VG participation and loneliness. Results from 18 studies were combined with meta-analysis being done for nine of them that used similar methodological approaches and measurement tools. The included studies represented 20,372 participants from 10 countries. The meta-analysis found that VG participation was positively and weakly associated with loneliness. The results of narrative synthesis revealed mixed findings on the relationship between VG participation and loneliness. Among cross-sectional studies, six studies found positive relationship, seven studies did not, and three studies found mixed relationship. The two longitudinal studies found that neither VG participation nor loneliness predicted each other 6 months later or 1-year later. The quality of evidence was rated very low for both cross-sectional and longitudinal studies. Overall, current literature base provides insufficient evidence to determine the relationship between VG participation and loneliness.

Tying back to the theoretical framework, individuals may choose to participate in VG to cope with loneliness, which provides them the sense of SP that can be converted into a resource that satisfies the relatedness tendency and reduces the feeling of loneliness. However, none of the studies included in this review found a negative relationship between VG participation and loneliness. The results from the meta-analysis even found a positive relationship. One possible explanation is that while video gaming provides opportunities for the players to interact with others who they usually do not know, high involvement in gaming reduces their time spent with significant others in real life, which may in turn increase their sense of loneliness as previous studies suggested (61). According to SP theory, computer-mediated communication typically produces a lesser perception of SP than face-to-face communication (62). The present systematic review supports this assumption and adds that participating in VG with less opportunities for face-to-face communication may have no influence on, or perhaps exacerbate, feelings of loneliness.

It is worth noting that in cross-sectional studies, the age of participants may be a significant factor in determining the association between VG participation and loneliness. Studies that recruited participants from universities or with an average age of over 18 years discovered a significant relationship between VG participation and loneliness (9, 43, 45, 49, 51), except for Jeong et al. (53). Meanwhile, studies recruiting participants from elementary through high schools or with an average age of less than 18 found no significant association between VG participation and loneliness (46, 47, 50, 54, 55), except for Yang and Liu (44). As a previous systematic review article demonstrated that feelings of loneliness tend to rise during emerging adulthood (63) and emerging adults spend predominant time in media use than doing any other activities (64), the finding of the current systematic review implies that while younger individuals may spend a substantial amount of time playing VG, they may also devote adequate amount of time communicating with others in real life, which contributes to an enhanced sense of SP. Older emerging adults who spend substantial amount of time playing VG may also choose to engage in other online activities, leading to a lack of sense of SP that may generate augmented sense of loneliness.

Although no study included in this systematic review examined the potential moderating effect of gender on the relationship between VG participation and loneliness, a substantial number of studies have revealed a gender difference in terms of loneliness, with males generally perceiving less loneliness than females (42, 46, 47, 50, 57). Additionally, Punamaki's study (50) observed a significant interaction between gender and information communication technology (ICT) usage (VG usage was used as one factor in calculating the ICT score), with more ICT usage being negatively associated with feelings of loneliness in males but not females, indicating that only males benefit from using ICT to alleviate their sense of loneliness. Thus, while the present systematic review cannot conclusively answer whether there is a gender difference in the link between VG participation and loneliness, it can be assumed that playing more VG is connected with a decreased perception of loneliness in males but not females. This hypothesis should be investigated further in future research.

The high heterogeneity in both meta-analysis and narrative synthesis across study findings is less likely because of the difference in measurement tools given the consistency in the loneliness and VG participation measurement. Rather, it is probably the result of less control of confounding variables. When separating passion into harmonious passion (an authentic and balanced relationship with the cherished activity) and obsessive passion [preoccupation and inflexible persistence toward the cherished activity (65)], Mandryk et al. (66) found that players with higher harmonious passion to play VG felt less loneliness, whereas players with higher obsessive passion to play VG had higher level of loneliness. The finding indicates the importance of different passion orientation in explaining the level of loneliness. One study found that there was a positive relationship between VG participation and loneliness for players playing other VGs than those playing Everquest II (42). This finding indicates game genres may have a moderating effect on the relationship between VG participation and loneliness. Kardefelt-Winther (67) found that while excessive online gaming was positively associated with loneliness, this relationship disappeared when controlling for self-perceived stress. Taken together, the high heterogeneity across studies may be explained by a variety of confounding variables that are not controlled in the studies included in this systematic review.

A study found that WoW players felt more lonely in real world than in online world (52). It is possible that VG players may feel more connected when they are online but feel more lonely when they are offline. In this review, the majority of the studies used the UCLA loneliness scale (68, 69) or its shortened version (70–72) to measure loneliness. Due to the fact that these scales do not require participants to think in a particular context when responding, it is reasonable to presume that participants tend to situate themselves in the real world rather than in the virtual world. Thus, the association between loneliness and VG participation addressed in the current systematic review may only be generalizable to real-world loneliness.

Several recommendations can be proposed in light of the findings of this systematic review. To begin, as none of the studies included in this systematic review discovered a negative association between VG participation and loneliness, practitioners and video game players should exercise caution when utilizing VGs to alleviate gamer's loneliness. Secondly, as loneliness has a variety of negative repercussions (11–14) and the association between VG participation and loneliness is yet unclear, video gamers should refrain from intensive VG participation in order to avoid unfavorable outcomes. Thirdly, because the current systematic review discovered that university students and emerging adults are more susceptible to the potentially VG-induced feelings of loneliness than their younger counterparts, more administrative and research attention should be directed toward preventing university students and emerging adults from engaging in excessive VG use in order to improve their overall mental health.

## 5. Limitations

First, a typical concern with systematic reviews is that the evidence synthesis is only as good as the articles included. The major limitation of this review is the very low quality of evidence on the relationship between VG participation and loneliness (73). Second, because insufficient number of studies was included in the meta-analysis [*n* < 10, (37)], sub-group analysis or meta-regression were not conducted to explain the high heterogeneity across studies. Third, this review did not include grey literature, abstracts, theses, conference proceedings, which may bias the results of this systematic review.

## 6. Future Research

This systematic review quantitatively and qualitatively synthesized the available evidence in the literature on the relationship between VG participation and loneliness. Given the inconsistency of the empirical results and the negative impacts of loneliness on well-being, more study in this area is strongly urged. To overcome the limitations existed in previous studies, several considerations are recommended. First, the potential causal association between VG participation and loneliness is unknown given to a dearth of experimental investigations in this field of research. Experimental evidence, particularly randomized controlled trials, would aid in our understanding of the possible causal association between VG and loneliness. Second, existing longitudinal studies used extended time intervals to examine the relationship between VG participation and loneliness. It is likely that individuals may have a brief time of increased loneliness following games and then return to their normal degree of loneliness. In future research, novel approaches such as ambulatory evaluation (74) may be employed to study the short-term effects between VG participation and loneliness. Third, because loneliness manifests differently in online and offline environments (52), future research should discriminate between these two forms of loneliness. This recommendation is crucial for intervention studies in which the primary objective is to alleviate loneliness (participants may experience a reduction of online loneliness but an increase in real-world loneliness following an intervention). Fourth, future study should examine the moderating effect of VG genres and gender gender difference on the association between loneliness and VG participation, as this field of unexplored. Fifth, all the previous studies collected VG participation data by asking participants to retrospectively recall on questions. Previous research has established that this data gathering strategy may introduce recall bias (59, 75). Future research may utilize the log file created by the game server to precisely quantify VG participation time.

## 7. Conclusion

The present study provides a systematic review on the relationship between VG participation and loneliness. The findings of narrative synthesis are inconclusive in terms of reaching a final conclusion on the relationship between VG participation and loneliness, despite a positive and weak relationship found in the meta-analysis. Overall, previous studies have very low quality of evidence. Additional studies are required to offer evidence for practitioners and policymakers in this area of research.

## Data Availability Statement

The original contributions presented in the study are included in the article/supplementary material, further inquiries can be directed to the corresponding author/s.

## Author Contributions

YL, TF, and DS contributed to conception of the study. YL, JM, and JX organized the database and selected appropriate articles. YL performed the statistical analysis and wrote the first draft of the manuscript. YL, MM, TF, and DS contributed to manuscript revision. All authors contributed to the article and approved the submitted version.

## Conflict of Interest

The authors declare that the research was conducted in the absence of any commercial or financial relationships that could be construed as a potential conflict of interest.

## Publisher's Note

All claims expressed in this article are solely those of the authors and do not necessarily represent those of their affiliated organizations, or those of the publisher, the editors and the reviewers. Any product that may be evaluated in this article, or claim that may be made by its manufacturer, is not guaranteed or endorsed by the publisher.

## References

[1] Newzoo. The Destination for Games Market Insights. Available online at: https://newzoo.com/ (accessed September 19, 2021).

[2] Raith L Bignill J Stavropoulos V Millear P Allen A Stallman HM . Massively multiplayer online games and well-being: a systematic literature review. Front Psychol. (2021) 12:2369. 10.3389/fpsyg.2021.698799 34276523 PMC8277937

[3] Prochnow T Patterson MS Hartnell L. Social support, depressive symptoms, and online gaming network communication. Ment Health Soc Inclus. (2020) 24:49–58. 10.1108/MHSI-11-2019-003324930050

[4] Maroney N Williams BJ Thomas A Skues J Moulding R. A stress-coping model of problem online video game use. International J Ment Health Addict. (2019) 17:845–58. 10.1007/s11469-018-9887-7

[5] Karaca S Karakoc A Gurkan OC Onan N Barlas GU. Investigation of the online game addiction level, sociodemographic characteristics and social anxiety as risk factors for online game addiction in middle school students. Commun Ment Health J. (2020) 56:830–8. 10.1007/s10597-019-00544-z 31907803

[6] Hanphitakphong P Thawinchai N Poomsalood S. Effect of prolonged continuous smartphone gaming on upper body postures and fatigue of the neck muscles in school students aged between 10-18 years. Cogent Eng. (2021) 8:1890368. 10.1080/23311916.2021.1890368

[7] Fortes LS De Lima-Junior D Fiorese L Nascimento-Júnior JRA Mortatti AL Ferreira MEC. The effect of smartphones and playing video games on decision-making in soccer players: a crossover and randomised study. J Sports Sci. (2020) 38:552–8. 10.1080/02640414.2020.1715181 31941416

[8] Hu H Zhang G Yang X Zhang H Lei L Wang P. Online gaming addiction and depressive symptoms among game players of the glory of the king in china: the mediating role of affect balance and the moderating role of flow experience. Int J Ment Health Addict. (2021). 1–14. 10.1007/s11469-021-00573-4

[9] Koban K Biehl J Bornemeier J Ohler P. Compensatory video gaming gaming behaviours and adverse outcomes and the moderating role of stress, social interaction anxiety, and loneliness. Behav Inform Technol. (2021). 1–18. 10.1080/0144929X.2021.1946154

[10] Heinrich LM Gullone E. The clinical significance of loneliness: a literature review. Clin Psychol Rev. (2006) 26:695–718. 10.1016/j.cpr.2006.04.002 16952717

[11] Peng A Tang Y He S Ji S Dong B Chen L. Association between loneliness, sleep behavior and quality: a propensity-score-matched case-control study. Sleep Med. (2021) 86:19–24. 10.1016/j.sleep.2021.08.008 34454179

[12] Lu M Wang R Lin H Pang F Chen X. Perceived social support and life satisfaction of Chinese parents of children with autism spectrum disorder: loneliness as a mediator and moderator. Res Autism Spectr Disord. (2021) 87:101829. 10.1016/j.rasd.2021.101829

[13] Nottage MK Oei NYL Wolters N Klein A Van der Heijde CM Vonk P . Loneliness mediates the association between insecure attachment and mental health among university students. Pers Individ Differ. (2022) 185:111233. 10.1016/j.paid.2021.111233

[14] Allan NP Volarov M Koscinski B Pizzonia KL Potter K Accorso C . Lonely, anxious, and uncertain: critical risk factors for suicidal desire during the COVID-19 pandemic. Psychiatry Res. (2021) 304:114144. 10.1016/j.psychres.2021.114144 34364010 PMC8442981

[15] Deci EL Ryan RM. Intrinsic Motivation and Self-Determination in Human Behavior. New York, NY; London: Plenum (1986). 10.1007/978-1-4899-2271-7

[16] Ryan RM Deci EL. Intrinsic and extrinsic motivation from a self-determination theory perspective: definitions, theory, practices, and future directions. Contemp Educ Psychol. (2020) 61:101860. 10.1016/j.cedpsych.2020.101860

[17] Lazarus RS Folkman S. Stress, Coping and Appraisal. New York, NY: Springer (1984).

[18] Compas BE Connor-Smith JK Saltzman H Thomsen AH Wadsworth ME. Coping with stress during childhood and adolescence: problems, progress, and potential in theory and research. Psychol Bull. (2001) 127:87–127. 10.1037/0033-2909.127.1.87 11271757

[19] King DL Delfabbro PH Zwaans T Kaptsis D. Clinical features and axis i comorbidity of Australian adolescent pathological internet and video game users. Austr N Z J Psychiatry. (2013) 47:1058–67. 10.1177/0004867413491159 23719181

[20] Griffiths M Kuss D King D. Video game addiction: past, present and future. Curr Psychiatry Rev. (2012) 8:308–18. 10.2174/157340012803520414

[21] Short J Williams E Christie B. The Social Psychology of Telecommunications. Toronto, ON; London; New York, NY: Wiley (1976).

[22] Lee KM. Presence, explicated. Commun Theory. (2004) 14:27–50. 10.1111/j.1468-2885.2004.tb00302.x

[23] Harms PC Biocca PF. Internal consistency and reliability of the networked minds measure of social presence. In: Alcaniz M, Rey B, editors. Seventh Annual International Workshop: Presence. Valencia: Universidad Politecnica de Valencia (2004).

[24] Biocca F Harms C Gregg J. The networked minds measure of social presence: pilot test of the factor structure and concurrent validity. In: 4th Annual International Workshop on Presence. Philadelphia, PA (2001). p. 1–9.

[25] Glasziou P Irwig L Bain C Colditz G. Systematic Reviews in Health Care: A Practical Guide. Cambridge: Cambridge University Press (2001). 10.1017/CBO9780511543500

[26] Swann C Keegan RJ Piggott D Crust L. A systematic review of the experience, occurrence, and controllability of flow states in elite sport. Psychol Sport Exerc. (2012) 13:807–19. 10.1016/j.psychsport.2012.05.006

[27] Page MJ McKenzie JE Bossuyt PM Boutron I Hoffmann TC Mulrow CD . The PRISMA 2020 statement: an updated guideline for reporting systematic reviews. BMJ. (2021) 372:n71. 10.1136/bmj.n71 33782057 PMC8005924

[28] Schardt C Adams MB Owens T Keitz S Fontelo P. Utilization of the PICO framework to improve searching PubMed for clinical questions. BMC Med Inform Decis Mak. (2007) 7:16. 10.1186/1472-6947-7-16 17573961 PMC1904193

[29] Pallavicini F Pepe A Minissi ME. Gaming in virtual reality: What changes in terms of usability, emotional response and sense of presence compared to non-immersive video games? Simul Gaming. (2019) 50:136–59. 10.1177/1046878119831420

[30] Hawkley LC Cacioppo JT. Loneliness matters: a theoretical and empirical review of consequences and mechanisms. Ann Behav Med. (2010) 40:218–27. 10.1007/s12160-010-9210-8 20652462 PMC3874845

[31] Leis O Lautenbach F. Psychological and physiological stress in non-competitive and competitive esports settings: a systematic review. Psychol Sport Exerc. (2020) 51:101738. 10.1016/j.psychsport.2020.101738

[32] Pels F Kleinert J. Loneliness and physical activity: a systematic review. Int Rev Sport Exerc Psychol. (2016) 9:231–60. 10.1080/1750984X.2016.1177849 PMC4706019 26807143

[33] Moola S Munn Z Tufanaru C Aromataris E Sears K Sfetcu R . Systematic reviews of etiology and risk. In: Aromataris E, Munn Z, editors. Joanna Briggs Institute Reviewer's Manual. The Joanna Briggs Institute. (2017). p. 5.

[34] Higgins JPT Altman DG Gøtzsche PC Jüni P Moher D Oxman AD . The Cochrane Collaboration's tool for assessing risk of bias in randomised trials. BMJ. (2011) 343:d5928. 10.1136/bmj.d5928 22008217 PMC3196245

[35] Guyatt G Oxman AD Akl EA Kunz R Vist G Brozek J . GRADE Guidelines: 1. introduction-GRADE evidence profiles and summary of findings tables. J Clin Epidemiol. (2011) 64:383–94. 10.1016/j.jclinepi.2010.04.026 21195583

[36] R Core Team. R: A Language and Environment for Statistical Computing [Manual]. Vienna: R Core Team (2021).

[37] Harrer M Cuijpers P Furukawa TA Ebert DD. Doing meta-analysis with R: a hands-on guide. Chapman and Hall/CRC (2021).

[38] Sterne JA Egger M Moher D. Addressing reporting biases. In: Sterne JAC, Moher MED, Boutron I, editors. Cochrane Handbook for Systematic Reviews of Interventions. John Wiley and Sons, Ltd (2008). p. 297–333. 10.1002/9780470712184.ch10

[39] Egger M Smith GD Schneider M Minder C. Bias in meta-analysis detected by a simple, graphical test. BMJ. (1997) 315:629–34. 10.1136/bmj.315.7109.629 9310563 PMC2127453

[40] Duval S Tweedie R. Trim and fill: a simple funnel-plot-based method of testing and adjusting for publication bias in meta-analysis. Biometrics. (2000) 56:455–63. 10.1111/j.0006-341X.2000.00455.x 10877304

[41] Schell R Hausknecht S Zhang F Kaufman D. Social benefits of playing wii bowling for older adults. Games Cult. (2016) 11:81–103. 10.1177/155541201560731326336883

[42] Shen C Williams D. Unpacking time online: connecting internet and massively multiplayer online game use with psychosocial well-being. Commun Res. (2011) 38:123–49. 10.1177/0093650210377196

[43] Zhou SX Leung L. Gratification, loneliness, leisure boredom, and self-esteem as predictors of SNS-game addiction and usage pattern among Chinese college students. Int J Cyber Behav Psychol Learn. (2012) 2:34–48. 10.4018/ijcbpl.2012100103

[44] Yang CC Liu D. Motives matter: motives for playing Pokémon go and implications for well-being. Cyberpsychol Behav Soc Netw. (2017) 20:52–7. 10.1089/cyber.2016.0562 28080150

[45] Wu AMS Lei LLM Ku L. Psychological needs, purpose in life, and problem video game playing among chinese young adults. Int J Psychol. (2013) 48:583–90. 10.1080/00207594.2012.658057 22506646

[46] Visser M Antheunis ML Schouten AP. Online communication and social well-being: how playing world of warcraft affects players' social competence and loneliness. J Appl Soc Psychol. (2013) 43:1508–17. 10.1111/jasp.12144

[47] Verheijen GP Burk WJ Stoltz SEMJ van den Berg YHM Cillessen AHN. Associations between different aspects of video game play behavior and adolescent adjustment. J Media Psychol. (2020) 32:27–39. 10.1027/1864-1105/a000253

[48] Sundberg M. Online gaming, loneliness and friendships among adolescents and adults with ASD. Comput Hum Behav. (2018) 79:105–10. 10.1016/j.chb.2017.10.020

[49] Snodgrass JG Dengah HJFI Polzer E Else R. Intensive online videogame involvement: a new global idiom of wellness and distress. Transcult Psychiatry. (2019) 56:748–74. 10.1177/1363461519844356 31084279

[50] Punamäki RL Wallenius M Hölttö H Nygård CH Rimpelä A. The associations between information and communication technology (ICT) and peer and parent relations in early adolescence. Int J Behav Dev. (2009) 33:556–64. 10.1177/0165025409343828

[51] Myrseth H Olsen OK Strand LA Borud EK. Gaming behavior among conscripts: the role of lower psychosocial well-being factors in explaining gaming addiction. Milit Psychol. (2017) 29:128–42. 10.1037/mil0000148

[52] Martončik M Lokša J. Do World of Warcraft (MMORPG) players experience less loneliness and social anxiety in online world (virtual environment) than in real world (offline)? Comput Hum Behav. (2016) 56:127–34. 10.1016/j.chb.2015.11.035

[53] Jeong SH Kim H Yum JY Hwang Y. What type of content are smartphone users addicted to?: SNS vs games. Comput Hum Behav. (2016) 54:10–7. 10.1016/j.chb.2015.07.035

[54] Cheung JCS Chan KHW Lui YW Tsui MS Chan C. Psychological well-being and adolescents' internet addiction: a school-based cross-sectional study in Hong Kong. Child Adolesc Soc Work J. (2018) 35:477–87. 10.1007/s10560-018-0543-7

[55] Buiza-Aguado C Alonso-Canovas A Conde-Mateos C Buiza-Navarrete JJ Gentile D. Problematic video gaming in a young Spanish population: association with psychosocial health. Cyberpsychol Behav Soc Netw. (2018) 21:388–94. 10.1089/cyber.2017.0599 29792521

[56] Ahn D Shin DH. Observers versus agents: divergent associations of video versus game use with empathy and social connectedness. Inform Technol People. (2016) 29:474–95. 10.1108/ITP-07-2014-0152

[57] Lemmens JS Valkenburg PM Peter J. Psychosocial causes and consequences of pathological gaming. Comput Hum Behav. (2011) 27:144–52. 10.1016/j.chb.2010.07.01524001297

[58] Kowert R Vogelgesang J Festl R Quandt T. Psychosocial causes and consequences of online video game play. Comput Hum Behav. (2015) 45:51–8. 10.1016/j.chb.2014.11.074

[59] Coughlin SS. Recall bias in epidemiologic studies. J Clin Epidemiol. (1990) 43:87–91. 10.1016/0895-4356(90)90060-3 2319285

[60] Nunnally JC. Psychometric Theory, 2nd Edn. New York, NY: McGraw-Hill book company (1978).

[61] Robbins JM DeLamater JD. Support from significant others and loneliness following induced abortion. Soc Psychiatry. (1985) 20:92–9. 10.1007/BF00594986 4002025

[62] Chang CM Hsu MH. Understanding the determinants of users' subjective well-being in social networking sites: an integration of social capital theory and social presence theory. Behav Inform Technol. (2016) 35:720–9. 10.1080/0144929X.2016.1141321

[63] Buecker S Mund M Chwastek S Sostmann M Luhmann M. Is loneliness in emerging adults increasing over time? A preregistered cross-temporal meta-analysis and systematic review. Psychol Bull. (2021) 147:787–805. 10.1037/bul0000332 34898234

[64] Coyne SM Padilla-Walker LM Howard E. Emerging in a digital world: a decade review of media use, effects, and gratifications in emerging adulthood. Emerg Adulthood. (2013) 1:125–37. 10.1177/2167696813479782

[65] Vallerand RJ Blanchard C Mageau GA Koestner R Ratelle C Léonard M . Les Passions de l'ame: on obsessive and harmonious passion. J Pers Soc Psychol. (2003) 85:756. 10.1037/0022-3514.85.4.756 14561128

[66] Mandryk RL Frommel J Armstrong A Johnson D. How passion for playing world of warcraft predicts in-game social capital, loneliness, and wellbeing. Front Psychol. (2020) 11:2165. 10.3389/fpsyg.2020.02165 33071843 PMC7533578

[67] Kardefelt-Winther D. Problematizing excessive online gaming and its psychological predictors. Comput Hum Behav. (2014) 31:118–22. 10.1016/j.chb.2013.10.017

[68] Russell D Peplau LA Ferguson ML. Developing a measure of loneliness. J Pers Assess. (1978) 42:290–4. 10.1207/s15327752jpa4203_11 660402

[69] Russell DW. UCLA loneliness scale (version 3): reliability, validity, and factor structure. J Pers Assess. (1996) 66:20–40. 10.1207/s15327752jpa6601_2 8576833

[70] Hays RD DiMatteo MR. A short-form measure of loneliness. J Pers Assess. (1987) 51:69–81. 10.1207/s15327752jpa5101_6 3572711

[71] Roberts RE Lewinsohn PM Seeley JR. A brief measure of loneliness suitable for use with adolescents. Psychol Rep. (1993) 72(3 Suppl.):1379–91. 10.2466/pr0.1993.72.3c.1379 8337350

[72] Valkenburg PM Peter J. Adolescents' identity experiments on the internet: consequences for social competence and self-concept unity. Commun Res. (2008) 35:208–31. 10.1177/0093650207313164

[73] Weir A Rabia S Ardern C. Trusting systematic reviews and meta-analyses: all that glitters is not gold!. Br J Sports Med. (2016) 50:1100–1. 10.1136/bjsports-2015-095896 26968215

[74] Himmelstein PH Woods WC Wright AGC. A comparison of signal- and event-contingent ambulatory assessment of interpersonal behavior and affect in social situations. Psychol Assess. (2019) 31:952–60. 10.1037/pas0000718 30958026 PMC6591090

[75] Raphael K. Recall bias: a proposal for assessment and control. Int J Epidemiol. (1987) 16:167–70. 10.1093/ije/16.2.167 3610443

